# Author Correction: Volatile resorption expedites eruption onset in large silicic systems

**DOI:** 10.1038/s41467-026-73472-8

**Published:** 2026-05-26

**Authors:** Franziska Keller, Meredith Townsend, Juliana Troch, Christian Huber

**Affiliations:** 1 Department of Earth and Environmental Sciences, Lehigh University, Bethlehem, PA USA; 2 Discipline of Geology, School of Natural Sciences, Trinity College Dublin, Dublin, Ireland; 3 Division of Geoscience and Geography, Faculty of Georesources and Materials Engineering, RWTH Aachen University, Aachen, Germany; 4 Department of Earth, Environmental, and Planetary Sciences, Brown University, Providence, RI USA

**Keywords:** Volcanology, Natural hazards, Geodynamics, Petrology

Correction to: *Nature Communications* 10.1038/s41467-026-70206-8, published online 12 March 2026

In the version of this article initially published, the two-tone gradient background in Fig. 1e appeared as a flat orange. The figure has been amended in the HTML and PDF versions of the article.

