# Supplementary material for: Volatile resorption expedites eruption onset in large silicic systems
Source: Nat Commun. 2026 Mar 12;17:3872. doi: 10.1038/s41467-026-70206-8 (PMC13125292; doi:10.1038/s41467-026-70206-8)
Supplement: Supplementary file 1 — Supplementary Information [file 41467_2026_70206_MOESM1_ESM.pdf]

Volatile resorption expedites eruption onset in large silicic systems

Franziska Keller[1/2*], Meredith Townsend[1], Juliana Troch[3], Christian Huber[4]

[1] Department of Earth and Environmental Sciences, Lehigh University, Bethlehem, PA, USA
[2] Discipline of Geology, School of Natural Sciences, Trinity College Dublin, Dublin, Ireland
[3] Faculty of Georesources and Material Sciences, RWTH Aachen University, Aachen, Germany
[4] Department of Earth, Environmental, and Planetary Sciences, Brown University, Providence, RI, USA

[*] Corresponding author. E-mail: kellerf@tcd.ie

Supplementary Information

Pages

Supplementary Figures .... 2 - 4

Supplementary Tables 4

Supplementary Methods .... 5 - 7

Supplementary Figures

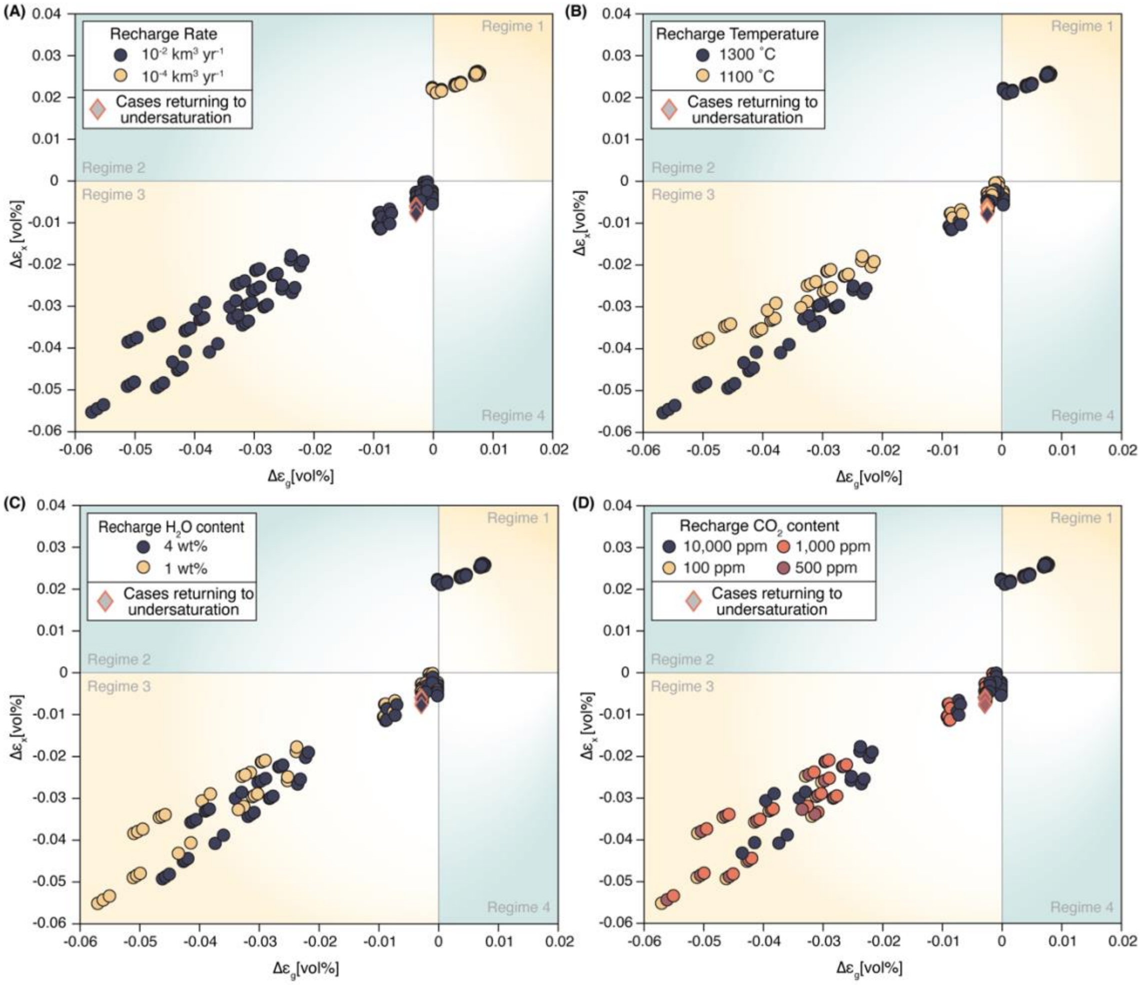

Supplementary Figure 1 – Variations in volatile and crystal fractions with recharge conditions. Changes in magmatic volatile phase (MVP) volume fraction ($\Delta\varepsilon_g$) and crystal volume fractions ($\Delta\varepsilon_x$) during Aso magma chamber simulations highlighting the impact of variable recharge conditions. All data are filtered for an initial MVP and diamond-shaped points with pink rims mark cases transitioning from volatile-saturated to volatile-undersaturated conditions during simulation.

(A) – (D) Subset of Aso data selected for comparison across recharge conditions: (A) recharge rates, (B) recharge temperatures, (C) recharge H_2O contents, and (D) recharge CO_2 contents.

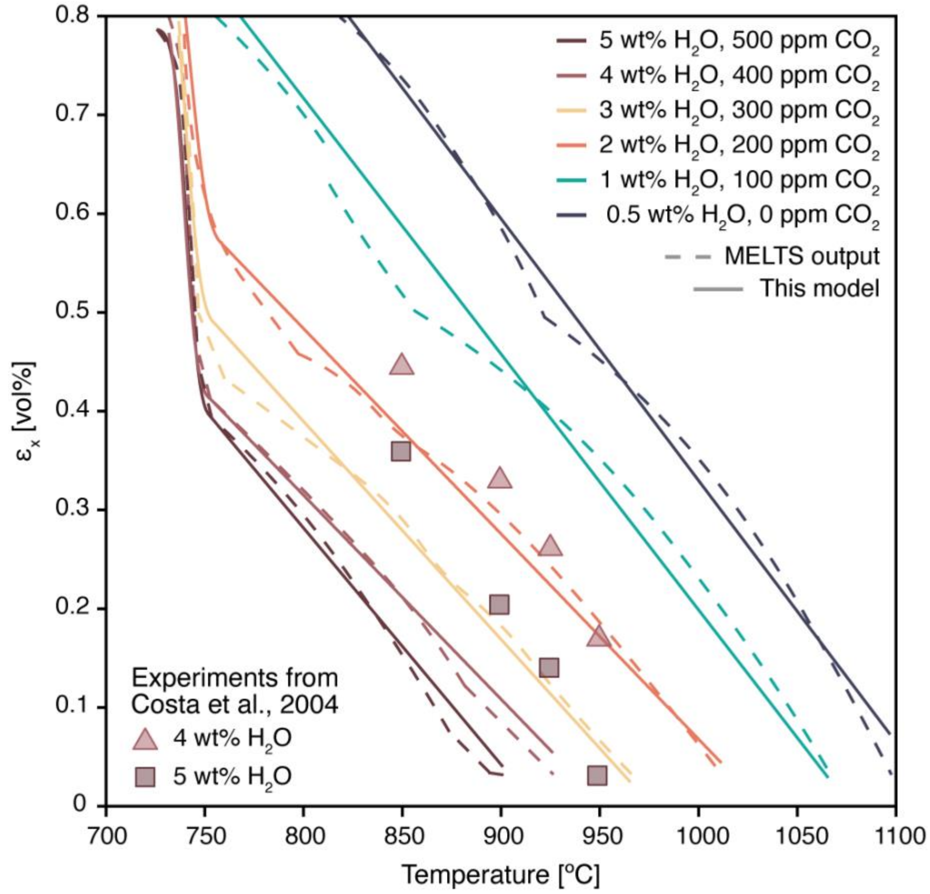

Supplementary Figure 2 – Model Calibration Curves.

Crystallinity-temperature curves from MELTS runs (dashed lines) compared with our parametrizations (solid lines) for variable H_2O (0.5 - 5 wt%) and CO_2 (0 - 500 ppm) contents at a constant pressure of 200 MPa. Triangles and squares represent experimental data from Costa et al. [61], triangles corresponding to runs with 4 wt% dissolved H_2O and square to runs with 5 wt% dissolved H_2O.

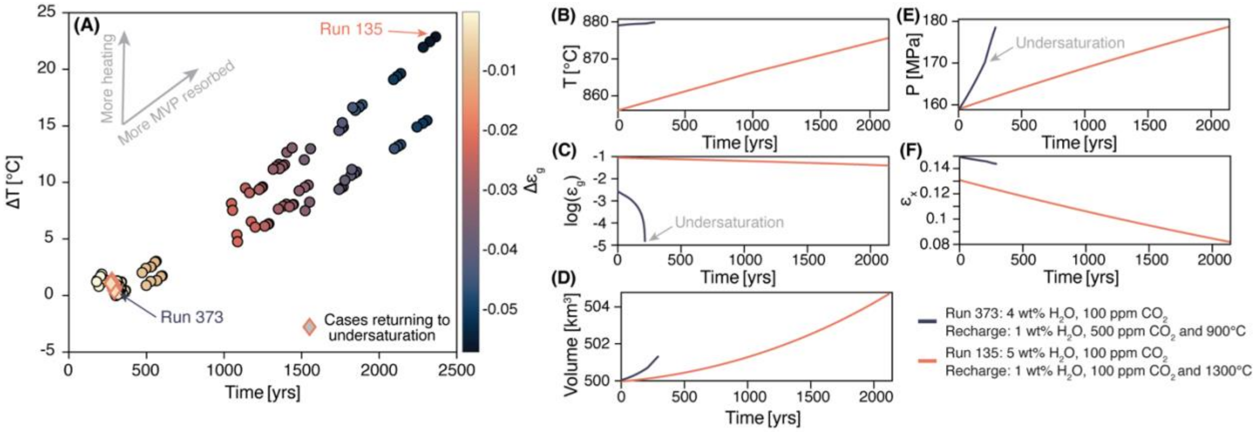

Supplementary Figure 3 – Variations of system parameters with time.
(A) Subset of Aso data with variable recharge conditions, showing changes in temperature (ΔT) as a function of time until eruption. Data points are filtered for an initial magmatic volatile phase (MVP) and color-coded by the total change in MVP volume fraction ($\Delta\varepsilon_g$) over the simulation time. All simulations were set up using Aso conditions summarized in Table 1. Diamond-shaped symbols with pink rims indicate cases that transitioned from volatile-saturated to volatile-undersaturated conditions during the simulation. Run 135 represents the longest simulation in this subset, while Run 373 corresponds to a short simulation undergoing full volatile resorption.
(B – F) Time evolution of key variables for Run 135 and Run 373 highlighted in (A): (B) temperature, (C) MVP volume fraction, (D) chamber volume, (E) pressure, and (F) crystal volume fraction.

Supplementary Table

SiO_2	TiO_2	Al_2O_3	FeO	MgO	CaO	Na_2O	K_2O
66.2	0.8	15.8	4.0	1.6	3.2	5.1	3.2

Supplementary Table 1. Aso-Y starting composition (in wt%) for Rhyolite MELTS [57] runs normalized to anhydrous.

Supplementary Methods: Calculate variations in volatile volume fractions using the conservation of water mass equation

To test the isolated effects of recharge-induced magma chamber pressurization (Process 1), magma mixing (Process 2), and variations in phase proportions (Process 3) on volatile resorption, we model the corresponding changes in magmatic volatile phase (MVP) volume fractions ($\Delta\varepsilon_g$) solving the equation for water mass conservation. We therefore compare the water mass prior to a recharge event ($M_{W,0}$) with the water mass after recharge entered the system ($M_{W,i}$) using the following equations:

$$M_{W,0} = \rho_m \varepsilon_{m,0} m_{eq,0} V_0 + \rho_{g,0} \varepsilon_{g,0} V_0 \quad (1)$$

$$M_{W,i} = \rho_m \varepsilon_{m,i} m_{eq,i} V_i + \rho_{g,i} \varepsilon_{g,i} V_i \quad (2)$$

We assume that crystals are nominally anhydrous and hence do not significantly contribute to water mass in the system, allowing us to neglect their contribution here. We further define the pre-recharge chamber to be at lithostatic pressure (P_{lit}), while the post-recharge chamber (P_i) is pressurized, with the overpressure (ΔP) reached during the recharge event being significantly smaller than P_{lit}:

$$P_0 = P_{lit} \quad (3)$$

$$P_i = P_{lit} + \Delta P \quad (4)$$

$$\Delta P = (P_i - P_0) \ll P_{lit} \quad (5)$$

The solubility of water (m_{eq}) and MVP density (ρ_g) are each a function of pressure and therefore post-recharge water solubility and MVP density can be expressed as follows:

$$m_{eq}(P_i) = m_{eq}(P_0) + \frac{\partial m_{eq}}{\partial P}(P_i - P_0) \quad (6)$$

$$\rho_g(P_i) = \rho_g(P_0) + \frac{\partial \rho_g}{\partial P}(P_i - P_0) \quad (7)$$

If we substitute these expressions into $M_{W,i}$ and consider that $\varepsilon_m + \varepsilon_x + \varepsilon_g = 1$, this gives the following equation to calculate water mass after the recharge event:

$$M_{W,i} = \rho_m (1 - \varepsilon_{x,i} - \varepsilon_{g,i})(m_{eq}(P_0) + \frac{\partial m_{eq}}{\partial P}\Delta P)V_i + (\rho_g(P_0) + \frac{\partial \rho_g}{\partial P}\Delta P)\varepsilon_{g,i} V_i \quad (8)$$

To calculate variations in the MVP volume fractions ($\Delta\varepsilon_g$) between the initial ($M_{W,0}$) and post-recharge ($M_{W,i}$) scenarios, we subtract the conservation for water mass equations from one another:

$$\frac{M_{W,i}}{V_i} - \frac{M_{W,0}}{V_0} = \rho_m (1 - \varepsilon_{x,i} - \varepsilon_{g,i}) m_{eq,0} + \frac{\partial m_{eq}}{\partial P}\Delta P) + (\rho_{g,0} + \frac{\partial \rho_g}{\partial P}\Delta P)\varepsilon_{g,i}$$

$$-\rho_m (1 - \varepsilon_{x,0} - \varepsilon_{g,0}) m_{eq,0} - \rho_{g,0} \varepsilon_{g,0} \quad (9)$$

Extending this equation and solving for $\Delta\varepsilon_g$ gives:

$$\Delta\varepsilon_g = \frac{\Delta P\left(\rho_m \frac{\partial m_{eq}}{\partial P}\left(1 - \varepsilon_{x,0} - \varepsilon_{g,0}\right) + \frac{\partial \rho_g}{\partial P}\varepsilon_{g,0}\right) - \Delta\varepsilon_x \rho_m \left(m_{eq,0} + \frac{\partial m_{eq}}{\partial P}\Delta P\right) - \left(\frac{M_{W,0}}{V_0} - \frac{M_{W,i}}{V_i}\right)}{\rho_m m_{eq,0} - \rho_{g,0} + \Delta P\left(\rho_m \frac{\partial m_{eq}}{\partial P} - \frac{\partial \rho_g}{\partial P}\right)} \tag{10}$$

To now simulate the isolated impact of recharge-induced pressurization (Process 1) and variations in melt and crystal phase proportions on $\Delta\varepsilon_g$ (Process 3), we assume closed system conditions where $M_{W,0} = M_{W,i}$ and $V_0 = V_i$. We then calculate variations in $\Delta\varepsilon_g$ with pressure (Process 1) by varying ΔP between 5 and 25 MPa, with $\Delta\varepsilon_x = 0$, $\varepsilon_{x,0} = 0.1$ and $\varepsilon_{g,0} = 0.05$, while keeping the remaining variables constant. To quantify the influence of variations in melt and crystal phase proportions on $\Delta\varepsilon_g$ (Process 3), we vary $\Delta\varepsilon_x$ between 0.01 and 0.05, corresponding to a decrease in crystallinity of 10% to 50% in the host magma assuming an initial crystallinity of 10 vol%. During these calculations ΔP is defined to equal 0 to isolate the impact of changes in the phase proportions alone. To ultimately calculate changes of $\Delta\varepsilon_g$ from mixing less volatile-rich magmas with volatile-enriched resident magmas (Process 2), we assume open system conditions allowing for variations in water mass and the total volume of the system. Therefore, we calculate the mass of H_2O and volume added to the system through recharge. Assuming an initial magma chamber volume of 500 km^3, we add up to 5 km^3 of recharge (representing 1% of the chamber volume) and calculate the associated mass of water using a recharge density of 2400 kg/m^3. To isolate the effects of changing water mass-to-volume ratio alone, we set $\Delta\varepsilon_x$ and ΔP to equal 0.

Symbols and constants used in here:

Symbol	Definition	Value or initial condition	Units
d	Depth of the chamber	6000	m
$\frac{\partial m_{eq}}{\partial P}$	Change of solubility with pressure	$2\text{x}10^{-10}$	$\frac{kg/m^3}{Pa}$
$\frac{\partial \rho_g}{\partial P}$	Change of exsolved volatiles density with pressure	$1.5\text{x}10^{-6}$	$\frac{kg/m^3}{Pa}$
$\varepsilon_m, \varepsilon_x, \varepsilon_g$	Volume fractions of melt, crystals, and exsolved volatiles	$\varepsilon_m + \varepsilon_x + \varepsilon_g = 1$	
$\Delta\varepsilon_x$, $\Delta\varepsilon_g$	Difference between crystal and exsolved volatiles volume fractions before and after recharge event	$\Delta\varepsilon_x = \varepsilon_{x,i} - \varepsilon_{x,0}$<br>$\Delta\varepsilon_g = \varepsilon_{g,i} - \varepsilon_{g,0}$	
g	Gravitational acceleration	9.81	m/s^2
$M_{W,0}$, $M_{W,i}$	Mass of water before and after the recharge event		kg
m_{eq}	Solubility of water in melt	parameterization of Liu et al., (2005)	wt%
P_0, P_i	Magma chamber pressure before and after the recharge event	$P_0 = P_{lit}$<br>$P_i = P_{lit} + \Delta P_c$	Pa
P_{lit}	Lithostatic pressure	$P_{lit} = \rho_r g d$	Pa
ΔP_c	Critical overpressure	20×10^6	Pa
ρ_m, ρ_x, ρ_g	Density of melt, crystals, and exsolved volatiles	$\rho_m = 2250$<br>$\rho_x = 2800$<br>$\rho_g = 350$	kg/m^3
ρ_r	Density of crust	2700	kg/m^3
V_0, V_i	Chamber volume before and after the recharge event		m^3
